# Microstructural Changes of Human Dentin Tubules after Citric Acid Immersion of Specimens Treated with Different Desensitising Approaches: An SEM Analysis

**DOI:** 10.3290/j.ohpd.b3464895

**Published:** 2022-10-19

**Authors:** Ogul Leman Tunar, Bahar Eren Kuru

**Affiliations:** a Assistant Professor, Yeditepe University, Faculty of Dentistry, Department of Periodontology,Istanbul, Turkey. Idea, experimental design, wrote the manuscript.; b Professor, Yeditepe University, Faculty of Dentistry, Department of Periodontology, Istanbul, Turkey. Hypothesis, proofread the manuscript, contribution substantially to discussion.

**Keywords:** acid immersion, dentinal hypersensitivity, Er:YAG laser, human dentin tubules, linear-oscillating device

## Abstract

**Purpose:**

This in-vitro study aimed to evaluate the effectiveness and acid resistance on dentin surfaces following the application of a linear-oscillating device (LOD) with a hydroxyapatite-based polishing fluid, Er:YAG laser or sodium fluoride (NaF) used alone or in combinations for desensitising purposes.

**Materials and Methods:**

Freshly extracted impacted third molars were used to obtain 120 specimens, all completely immersed in 1% citric acid for 5 min and divided randomly into 6 groups. Group I, with no treatment, served as the control; group II: Er:YAG laser (30 Hz, 60 mJ/pulse, 10 s); group III: NaF gel; group IV: LOD; groups V and VI: the combinations of NaF+Er:YAG and LOD+Er:YAG, respectively, were applied. Following these treatments, the effectiveness of each was evaluated in half of the specimens in each group (n = 10). The other half of the specimens (n = 10) served for acid-resistance testing. All evaluations were made on SEM photomicrographs.

**Results:**

The post-treatment tubule diameters and numbers were the lowest with LOD+Er:YAG, followed by NaF+Er:YAG, LOD, Er:YAG and NaF. Paired comparisons revealed LOD+Er:YAG to be the best treatment method (p < 0.05). After 3 h of acid immersion, all treatments revealed significant increases (p < 0.05) in both tubule number and diameter study between post-treatment and post-acid immersion values. The exception was LOD+Er:YAG. LOD+Er:YAG showed the highest resistance to acid challenge, presenting the lowest increase in tubular diameters and numbers followed by NaF+Er:YAG, LOD, Er:YAG and NaF.

**Conclusion:**

Combined LOD+Er:YAG treatment revealed the highest effectiveness and acid resistance. Further clinical studies are warranted to confirm these in-vitro results.

Dentin hypersensitivity (DH) is the most frequently encountered but underrated problem in dental practice. DH can be described as a painful condition arising from the response to chemical, thermal, tactile, or osmotic stimuli. DH cannot be ascribed to any other dental defect or disease except exposed dentin surfaces. The prevalence of DH varies from 3% to 98%, and mostly affects patients in their 30s or 40s.^3,4,13^ The aetiology of DH is multifactorial, although the best-documented factors are erosion from dietary substances and gingival recession caused by periodontal disease or traumatic toothbrushing.^1,15,17,22,45^ According to a consensus-based recommendation, researchers have concluded that acid erosion and gingival recession, rather than traumatic cervical enamel loss, are the most important key factors causing DH.^12^

Loss of mineralised tooth structure is called erosion and occurs during acid attacks due to two different sources: intrinsic (e.g. gastroesophageal reflux) and extrinsic (e.g. acidic beverages).^18^ With the global increase of fast-food consumption, not only gastric problems but also the acidic environment of the oral cavity appear to lower the pH level, exposing and enlarging the dentinal tubules through erosive effects. Normal toothbrushing cannot cause significant enamel loss or gingival recession.^47^ On the other hand, erosion from acidic foods and drinks in combination with normal toothbrushing may result in significant tooth wear on any aspect of the tooth surface, but especially in the cervical areas, and result in DH.^50^ Brannström’s hydrodynamic theory^6^ is the most well-documented theory to explain DH. According to this theory, a pain-triggering stimulus (tactile, osmotic, or chemical) changes the fluid flow in the exposed dentinal tubules, which causes a change of the pressure in the pulp. This mechanoreceptor effect stimulates the pulpal nerve endings and causes pain. In-vitro studies have shown that teeth with hypersensitivity present a high number of open dentinal tubules and enlarged tubular orificies.^2,49^

There are numerous DH treatment methods. A variety of chemical and physical agents have been used, including tubule occluding agents (e.g. potassium oxalate, sodium fluoride [NaF]),^42,48^ protein precipitants (e.g. glutaraldehyde)^31^ and tubule sealants^30^ or lasers,^16^ all of which aim at preventing dentinal fluid movement. Laser systems such as Er:YAG, CO_2_, Nd:YAG, He-Ne, GaAlAs, and Er,Cr:YSGG have been used for DH treatment for the past 4 decades.^11,19,23,41,42^ The Er:YAG laser can be used safely without thermally damaging the dental hard/soft tissues due to its thermomechanical ablation ability, patient-friendly mechanism of action, and a wavelength that is well absorbed in water but less so in hydroxyapatite.^43^ The rationale of tubule occluding agents is to block or minimise the dentinal tubule orifices, which reduces or eliminates the dynamic pressure changes from external stimuli. Today, the most commonly used tubule occluding chemical agent in dental practice is still professionally-applied NaF gel. Although many studies have been conducted with all these agents,^23,29,37^ the immediate and long-term pain reduction are still questionable and under debate.

A new-generation ultrasonic system with linear oscillation became available around the year 2000. Its action is a non-elliptical movement of the tip. This results in minimal invasiveness for safe and comfortable mechanical, nonsurgical periodontal therapy.^8,14,20^ In addition to water, the device can be applied with an adjunctive polishing fluid containing 10-μm hydroxyapatite (HA) granules. A limited number of studies have shown that this fluid may block DH symptoms.^7^ However, to the best of our knowledge, no study to date has investigated or confirmed the potential DH desensitising effects or their longevity in an acid environment of this linear oscillating device (LOD) with an adjunctive polishing fluid containing 10-μm HA granules.

It has been reported that the single use of many therapeutic agents is insufficient in the elimination or control of DH,^21^ thus causing recurrence of the pain symptoms. For this reason, researchers have attempted to combine therapies for improving the effectiveness and the longevity of the treatment results.^11,21,41^ The results are promising for combined approaches when different chemical agents and physical devices are used in combination.^21,23,25,41^ However, although it is of critical importance to evaluate the resistance of the occluded dentinal tubules, only a few studies exist on the resistance to acid challenges after different treatments.^27^

Therefore, this multi-armed in-vitro study aimed to investigate both the tubular occlusion and thereafter the acid resistance after the application of single and combined desensitizing procedures, including NaF gel, Er:YAG laser and LOD applied to citric acid-opened human dentinal tubules. The study hypothesis is that the LOD system, as a mechanical, nonsurgical treatment method, is also effective in occluding open dentinal tubules and promoting resistance to acid attacks.

## Materials and Methods

This study was approved by the scientific committee of Yeditepe University Faculty of Dentistry (354/09/2020) and the ethics board of Yeditepe University (KAEK: 1425/2021). The sample size calculation was performed based on a previous DH study.^44^ According to this calculation, α was 0.05, the power of the test (b) was 80% and the effect size (Cohen’s d) was 0.05 for the number of dentinal tubules. The calculated sample number was found to be a minimum of 7 in each study group.

### Preparation of Dentin Specimens

Sixty freshly extracted, impacted human mandibular third molars were obtained from adults aged between 24–32 years. The extracted teeth were prepared as described in previous studies.^11,41^ To obtain the 3-mm-thick experimental dentin specimens from the extracted tooth, two horizontal cuts were made perpendicular to the long axis of the tooth, one of which was made through the cementoenamel junction and the other 3 mm below the first cut. Thereafter, the obtained piece was divided into halves with a third transverse cut, and the halves were subsequently coded. In this way, a total of 120 specimens were obtained. The specimens were placed on a coin-sized (19.25 mm x 2 mm) cast filled with acrylic resin in order to transfer the codes, standardize the working position, and facilitate the ease of application. Special care was taken ensure that the resin did not cover or contact the cementum surface of the specimen. In the next step, to remove the cementum layer, the specimen surfaces were wet polished with a sequence of carbide papers (600-, 1200-, and 2000-grit) to expose the dentin. The specimens were then placed in an ultrasonic cleaner containing distilled water and washed for 5 min.

### Experimental Groups and Treatments

The specimens were divided into 6 groups according to a randomisation table (randomlists.com). After group assignment, specimens were completely immersed in a 1% citric acid solution for 5 min to remove the smear layer and simulate root surfaces affected by DH, then rinsed with distilled water and air dried. Desensitizing treatment procedures were then performed in the test groups. The groups are described below.

- Group I (control) (n = 20): No treatment was applied to these specimens to represent the pre-treatment phase.
- Group II (Er:YAG) (n = 20): The specimens were irradiated with Er:YAG laser (DE-Light, Hoya ConBio; Fremont, CA, USA) using a 60-μm–diameter chisel quartz tip at an energy level of 60 mJ per pulse, and a repetition rate of 30 Hz, for 10 s.^21^ The laser beam was moved in a mesiodistal direction with the beam directed perpendicularly to the dentin surface in non-contact mode without water irrigation at a distance of 3–4 mm.
- Group III (NaF) (n = 20): A topical NaF gel (Enamel Pro Gel, Premier Dental Products; Plymouth Meeting, PA, USA) was applied to the specimens with a bonding brush for 4 min and then gently rinsed with distilled water.^11^
- Group IV (LOD) (n = 20): The LOD system (Vector Paro Pro, Dürr Dental SE; Bietigheim-Bissingen, Germany) was applied with a straight tip (Paro probe straight tip, Dürr Dental SE) in combination with the hydroxyapatite-based polishing fluid (Vector Fluid Polish, Dürr Dental SE) on the specimens. The surfaces of the specimens were traced with a sweeping motion and the tip was kept in constant motion over the working area. The application period was 30 s at 80% operating power.
- Group V (NaF+Er:YAG) (n = 20): Following the application of topical NaF gel for 4 min, the specimens were gently rinsed with distilled water and then irradiated with Er:YAG laser at the same parameters and in the same fashion as in Group II.
- Group VI (LOD+Er:YAG) (n = 20): Following the application of the LOD system, the specimens were then irradiated with Er:YAG laser at the same parameters and in the same fashion as in Groups II and IV.

**Fig 1:**
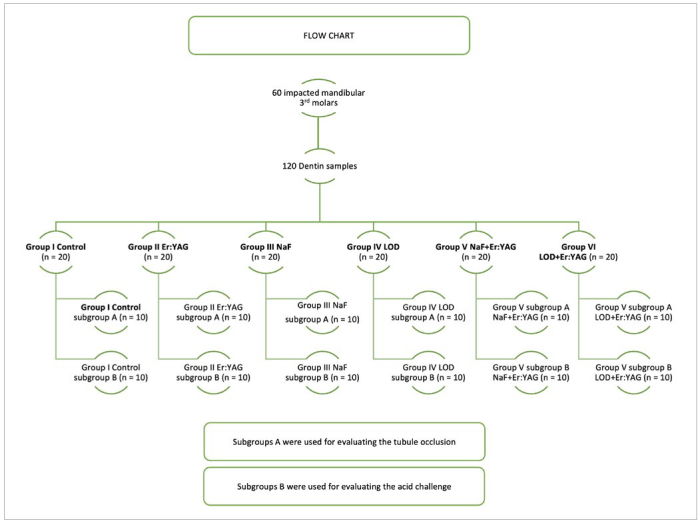
Flow chart of the study.

The first 10 specimens of the test groups were used for evaluating the tubule occlusion, whereas the other 10 were submitted to an acid challenge, simulating the continuation of an acidic diet. Therefore, the groups were divided into two subgroups, A and B, leaving 10 specimens in each for two different evaluation purposes (Fig 1). Since a specific sample coating is required for SEM analysis of tubule occlusion, the suitability of the specimens for the acid challenge assessment was precluded due to this coating. Therefore, the remaining 10 specimens in each group were used for evaluation after the acid challenge. The specimens of subgroup A were directly prepared for tubule occlusion assessment and fixed in 2.5% glutaraldehyde, placed in 0.1M phosphate-buffered saline (pH 7.2) for 24 h at room temperature, washed with distilled water, and air dried. For the acid challenge, subgroup B specimens in each group were first completely immersed in 1% citric acid for 3 h, washed with distilled water, and then fixed in the same manner as described above for subgroup A.

### SEM Analysis and Measurements

For the SEM (JSM 6335F; JEOL-USA; Peabody, MA, USA) analysis of the microstructural changes on dentinal surfaces, the specimens were coated with an approximately 20-nm-thick platinum layer, and photomicrographs of each specimen were taken at 1000X and 2000X magnifications at an accelerating voltage of 10-15 kV.

Selection of the fields for quantitative evaluation was standardised as follows: the intersection of the two diagonal lines at the midpoint of the sample surface was located at 20X magnification and thereafter the intersection point was further zoomed in on, to obtain photomicrographs under higher magnifications of 1000X and 2000X.^40^

The photomicrographs were then uploaded into a software program (Image J) for accurate measurements. The number of dentinal tubules in each specimen were counted on the 1000X images. On the 2000X images, the diameters of the dentinal tubule orifices were determined. Only circular tubule orifices were evaluated; elliptical tubules reflected a misleading oblique cut and were excluded to minimise measurement errors. The number and the diameter of dentinal tubules determined on the photomicrographs were adjusted to the actual size using the reference scale bar on the image. All morphometric measurements for the number and diameters of tubules were performed by a single investigator (OLT) (ICC: 1.000; 95% CI: 1.000–1.000) (ICC: 0.999; 95% CI: 0.999–1.000).

### Statistical Analysis

The data were analysed using IBM SPSS Statistics 22 (IBM; Armonk, NY, USA). A non-normal distribution was found by the Shapiro-Wilks test. The Kruskal-Wallis test was used for multiple comparisons of the parameters between the groups, and Dunn’s test was used to determine pairwise significance. The Wilcoxon signed-rank test was used for intragroup comparisons. Statistical significance was set at p < 0.05.

## Results

**Fig 2:**
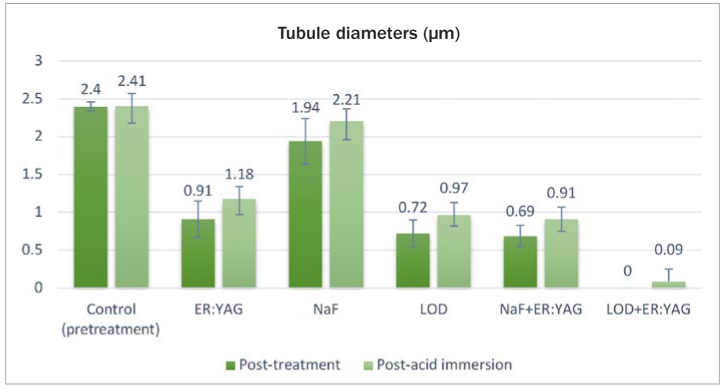
Post-treatment and post-acid immersion tubule diameters of all groups.

**Fig 3:**
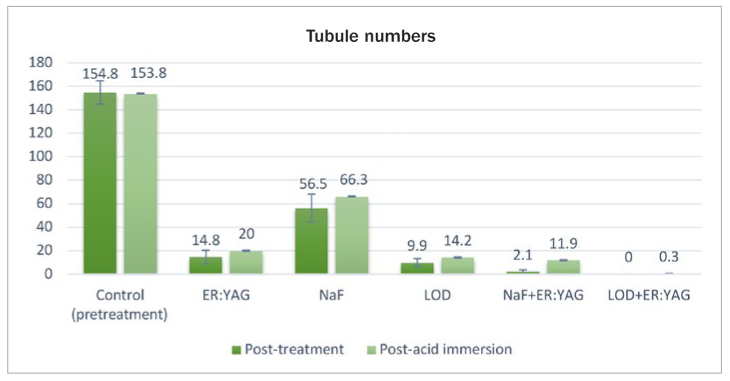
Post-treatment and post-acid immersion tubule numbers of all groups.

According to the comparisons with the pre-treatment values (control A) as well as between the treatment groups, post-treatment tubule diameters and numbers with LOD+Er:YAG application (0 ± 0 µm; 0 ± 0) were the lowest, followed by NaF+Er:YAG (0.69 ± 0.14 µm; 2.10 ± 1.66), LOD (0.72 ± 0.18 µm; 9.90 ± 3.38), Er:YAG laser (0.91 ± 0.24 µm; 14.8 ± 5.69) and NaF gel (1.94 µm ± 0.30; 56.5 ± 11.82) (Tables 1 and 2) (Figs 2 and 3). In pairwise comparisons, LOD+Er:YAG application was found to be the best treatment method in terms of reducing the tubule diameters (p < 0.05). However, the pairwise comparison of reduction in tubule numbers showed no statistically significant difference between the LOD+Er:YAG and the NaF+Er:YAG group (Tables 1a, 2a).

**Table 1:** Evaluation of post-treatment and acid immersion tubule diameter values

	Tubule diameters (µm)		p**
	Post-treatment	Post-acid immersion	
	Mean ± SD	Mean ± SD	
Control (pretreatment)	2.40 ± 0.06	2.41 ± 0.07	0.735
Er:YAG	0.91 ± 0.24	1.18 ± 0.23	0.022^ǂ^
NaF	1.94 ± 0.30	2.21 ± 0.21	0.049^ǂ^
LOD	0.72 ± 0.18	0.97 ± 0.25	0.013^ǂ^
NaF+Er:YAG	0.69 ± 0.14	0.91 ± 0.15	0.013^ǂ^
LOD+Er:YAG	0 ± 0	0.09 ± 0.16	0.109
*p	0.000^ǂ^	0.000^ǂ^	

*Kruskal-Wallis test; **Wilcoxon signed-rank test; ^ǂ^p < 0.05.

**Table 2:** Evaluation of post-treatment and post-acid immersion tubule numbers

	Tubule numbers		p**
	Post-treatment	Post-acid immersion	
	Mean ± SD	Mean ± SD	
Control (pretreatment)	154.8 ± 9.9	153.8 ± 7.6	0.684
Er:YAG	14.8 ± 5.69	20.0 ± 5.68	0.011^ǂ^
NaF	56.5 ± 11.82	66.3 ± 11.78	0.008^ǂ^
LOD	9.9 ± 3.38	14.2 ± 2.39	0.018^ǂ^
NaF+Er:YAG	2.1 ± 1.66	11.9 ± 6.47	0.007^ǂ^
LOD+ERYAG	0 ± 0	0.3 ± 0.48	0.083
p*	0.000^ǂ^	0.000^ǂ^	

*Kruskal-Wallis Test; **Wilcoxon signed-rank test; ^ǂ^p < 0.05.

**Table 1a:** Pair-wise comparison of the post-treatment tubule diameters

Control (pretreatment)	Control (pretreatment)					
Er:YAG	0.022*	Er:YAG				
NaF	1.000	0.046*	NaF			
LOD	0.001*	0.415	0.007*	LOD		
NaF+Er:YAG	0.000*	0.240	0.033*	0.719	NaF+Er:YAG	
LOD+ERYAG	0.000*	0.018*	0.000*	0.016*	0.039*	LOD+Er:YAG

Dunn’s test, *p < 0.05.

**Table 2a:** Pair-wise comparison of the post-treatment tubule numbers

Control (pretreatment)	Control (pretreatment)					
Er:YAG	0.004*	Er:YAG				
NaF	0.198	0.040*	NaF			
LOD	0.005*	0.452	0.021*	LOD		
NaF+Er:YAG	0.000*	0.015*	0.001*	0.095	NaF+Er:YAG	
LOD+ERYAG	0.001*	0.008*	0.001*	0.007*	0.301	LOD+Er:YAG

Dunn’s test, *p < 0.05.

After 3 h of acid immersion (post-acid immersion), all treatments produced statistically significant changes (p < 0.05) in both study parameters within each group between post-treatment and post-acid immersion values, representing increases in tubule diameter and number, except for the LOD+Er:YAG group (Tables 1 and 2) (Figs 2 and 3). In other words, there was no statistically significant difference within the LOD+Er:YAG group between post-treatment and post-acid immersion. The LOD+Er:YAG group showed the highest resistance to acid attack, with the lowest increase in tubule diameters and numbers (0.09 ± 0.16 µm; 0.3 ± 0.48), followed by the groups of NaF+Er:YAG (0.91 ± 0.15 µm; 11.9 ± 6.47), LOD (0.97 ± 0.25 µm; 14.2 ± 2.39), Er:YAG laser (1.18 ± 0.23 µm; 20.0 ± 5.68) and NaF gel (2.21 ± 0.21 µm; 66.3 ± 11.78) (Tables 1b and 2b).

**Table 1b:** Pair-wise comparison of the post-acid immersion tubule diameters

Control (pretreatment)	Control (pretreatment)					
Er:YAG	0.004*	Er:YAG				
NaF	1.000	0.042*	NaF			
LOD	0.002*	0.330	0.039*	LOD		
NaF+Er:YAG	0.000*	1.000	0.012*	1.000	NaF+Er:YAG	
LOD+ERYAG	0.000*	0.013*	0.000*	0.019*	0.044*	LOD+Er:YAG

Dunn’s test, *p < 0.05.

**Table 2b:** Pair-wise comparison of the post-acid immersion tubule numbers

Control (pretreatment)	Control (pretreatment)					
Er:YAG	0.003*	Er:YAG				
NaF	0.200	0.036*	NaF			
LOD	0.000*	0.228	0.003*	LOD		
NaF+Er:YAG	0.000*	0.176	0.002*	0.883	NaF+Er:YAG	
LOD+ERYAG	0.000*	0.001*	0.001*	0.029*	0.041*	LOD+Er:YAG

Dunn’s test, *p < 0.05.

**Fig 4:**
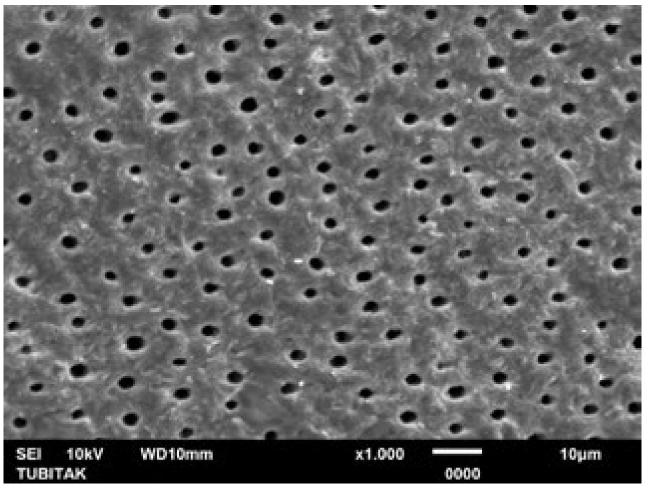
Representative photomicrograph of the pretreatment control (citric acid) group (1000X).

**Fig 5:**
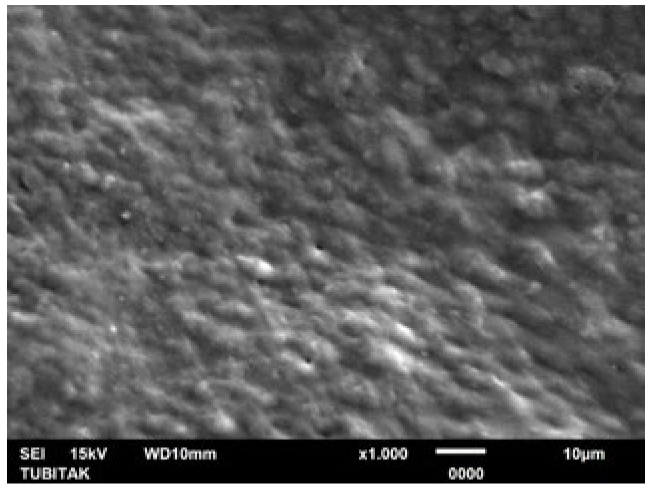
Representative photomicrograph of the Er:YAG laser group (post-treatment, 1000X).

**Fig 6:**
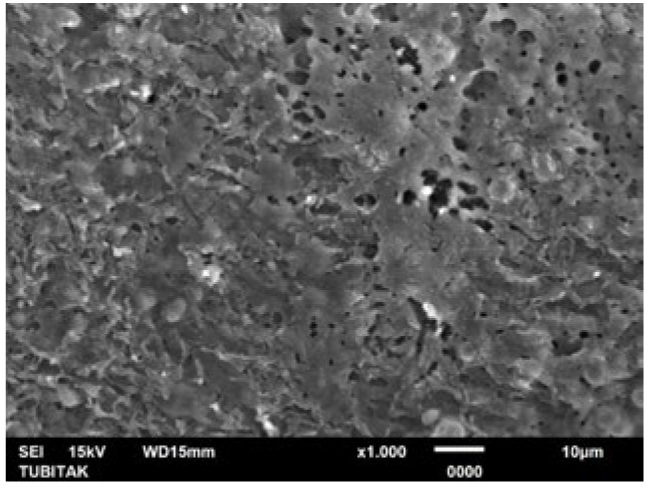
Representative photomicrograph of the Er:YAG laser group (post-acid immersion, 1000X).

**Fig 7:**
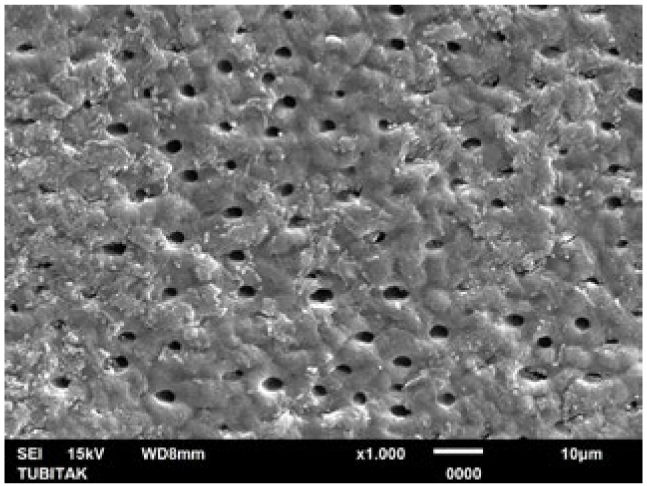
Representative photomicrograph of the NaF group (post-treatment, 1000X).

**Fig 8:**
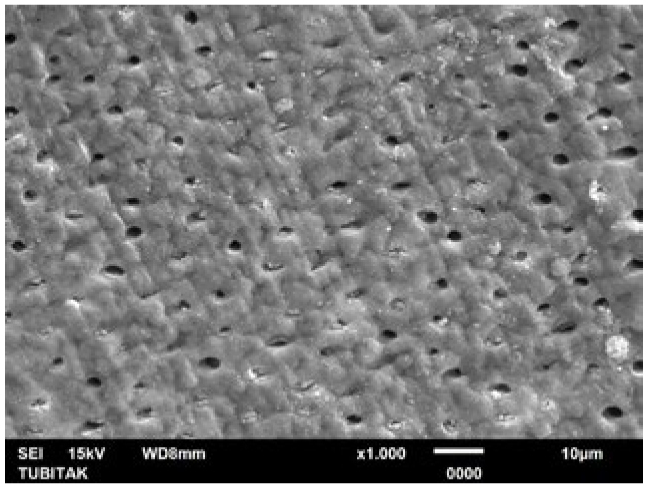
Representative photomicrograph of the NaF group (post-acid immersion, 1000X).

**Fig 9:**
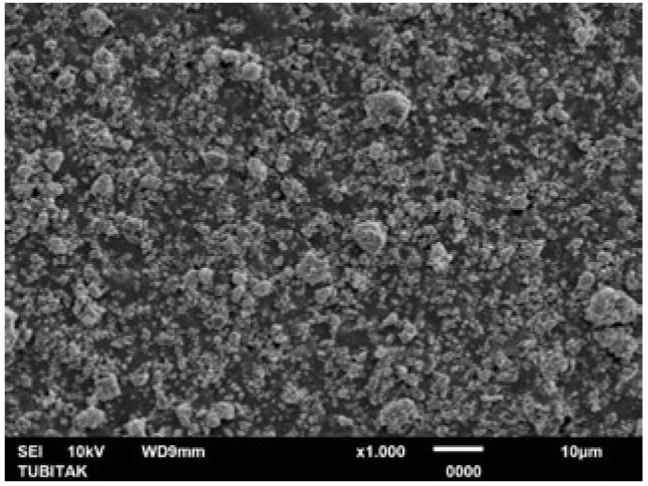
Representative photomicrograph of the LOD group (post-treatment, 1000X).

**Fig 10:**
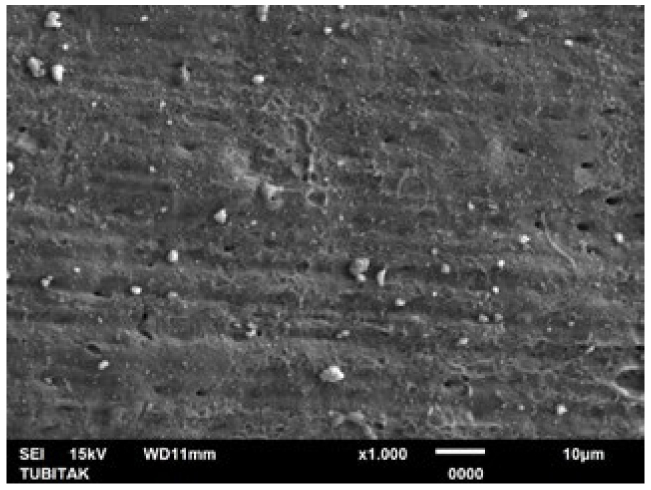
Representative photomicrograph of the LOD group (post-acid immersion, 1000X).

**Fig 11:**
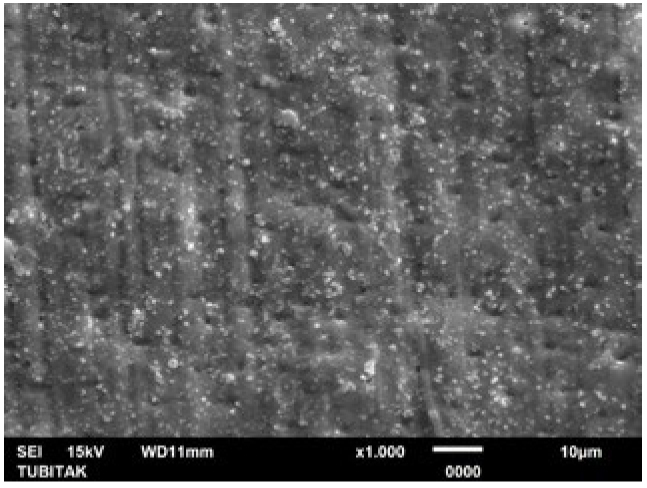
Representative photomicrograph of the NaF+Er:YAG group (post-treatment, 1000X).

**Fig 12:**
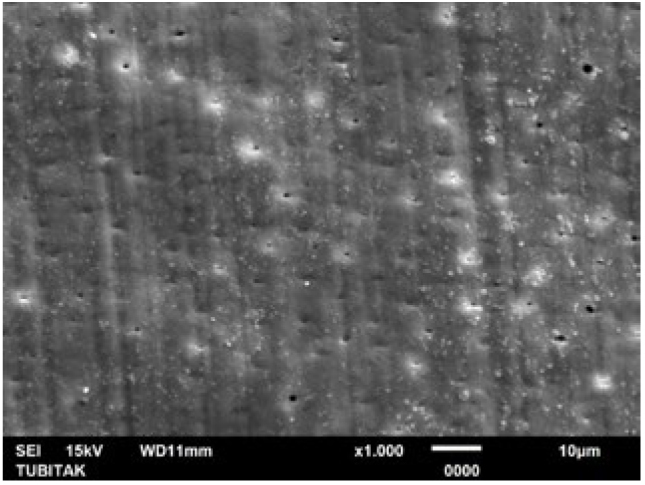
Representative photomicrograph of the NaF+Er:YAG group (post-acid immersion, 1000X).

**Fig 13:**
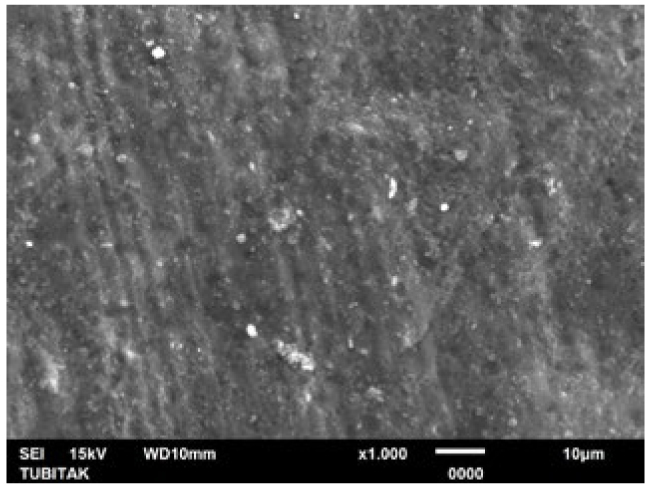
Representative photomicrograph of the LOD+Er:YAG group (post-treatment, 1000X).

**Fig 14:**
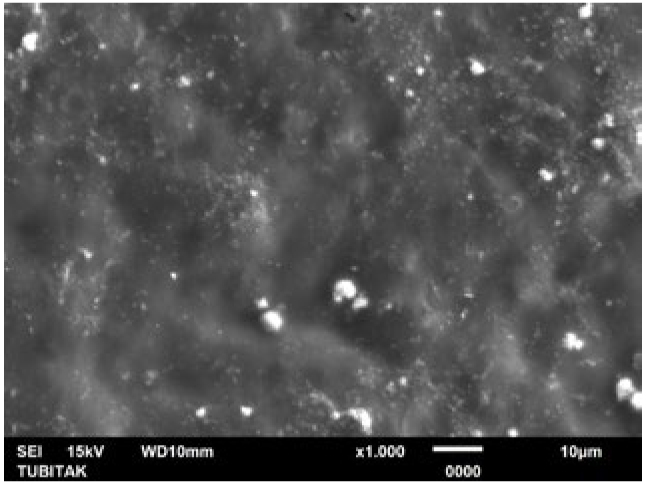
Representative photomicrograph of the LOD+Er:YAG group (post-acid immersion, 1000X).

LOD treatment alone was found to be as effective as the NaF+Er:YAG combination and Er:YAG laser in occluding the tubules. There were no statistically statistical differences in post-treatment tubule diameters and numbers when LOD treatment was compared with the NaF+Er:YAG combination and Er:YAG laser alone in pairs (Tables 1a and 2a). Similarly, regarding the post-acid immersion results, LOD treatment again yielded no statistically significant differences compared to the NaF+Er:YAG combination and Er:YAG laser alone in terms of tubule diameters and numbers. This demonstrates that the acid resistance after LOD treatment alone is as high as in the aforementioned groups. Representative SEM images of the groups are shown in Figs 4-14.

## Discussion

DH, considered a real clinical problem for periodontal patients with gingival recessions and exposed root surfaces,^36^ causes sharp pain and discomfort after any thermal, osmotic, or chemical stimulus and considerably affects the quality of life. Moreover, the longevity of dentinal tubule occlusion in the oral cavity is also critically important for improving the patient’s quality of life, which is deemed the main objective of any treatment. Unfortunately, despite many approaches, a universally accepted treatment option to treat profound DH symptoms is lacking, and thus further studies are needed.

The present in-vitro study, a comparative evaluation of an ultrasonic device (LOD, specifically designed for nonsurgical treatment) used together with a fluid containing HA vs other methods, investigated the effects on the reduction of dentin tubule diameter and numbers as well as the resistance of post-treatment tubule occlusion to an acid attack. DH is a difficult-to-manage clinical problem, characterised by a large number of open dentinal tubules exposed to the oral environment. The treatment principle is occlusion of the tubules to ease pain and patient discomfort.

It is known that the application of individual treatment approaches for DH are less effective than combined modalities.^11,41^ Therefore, besides the single approaches such as NaF gel, Er:YAG laser or LOD alone, combinations of NaF+Er:YAG and LOD+Er:YAG were also included in our multi-armed study design due to possible synergistic effects. Many studies have used professionally applied NaF products^32,48^ and laser treatment^24^ to promote dentinal tubule occlusion and prevent dentinal fluid movement. NaF application, an established procedure in the prevention of demineralisation of dental hard tissues,^38,39^ is associated with the formation of a calcium fluoride-like precipitate leading to the occlusion of dentinal tubules.^39^ The mechanism of tubule occlusion by dental lasers is through melting and sealing of the open dentinal tubules. The type of laser and the parameters used in this study were chosen based on our previous clinical and in-vitro studies.^11,21,41^

The LOD system, with its uncommon working principle of deflecting a horizontal oscillation vertically, has been discussed in recent years.^7-10,34,35^ Our hypothesis about the expected possible effects of the LOD system on DH emerged from results of the limited number of available studies, which reported root surface characteristics and patient perceptions of low pain intensity.^7-10,33^ In the LOD system, effective mechanical instrumentation was achieved with a low level of pain. The use of adjunctive HA fluid during debridement is recommended for the patient’s comfort and for the prevention of root substance removal. Since the LOD system was originally presented as a nonsurgical treatment method, researchers primarily evaluated the mechanical effects of nonsurgical treatment. Overall, the available studies revealed many positive results, such as clinical improvements as well as reduced patient discomfort and pain during instrumentation.^10,20^ On the other hand, the in-vitro study by Braun et al,^7^ which compared the use of the device with water vs HA-containing polishing fluid, investigated the presence and thickness of the smear layer as well as alterations on the root surfaces, showing a 2-µm granular layer covering the dentin surfaces only in the HA-fluid group. No further details were given in that study. In their in-vivo study, Schwarz et al^34^ stated that root surfaces treated with the LOD system generally exhibited a smooth, homogeneous appearance with slight superficial irregularities, lacking crater formation or damage. For this novel in-vitro study, we compiled clinical and in-vitro/in-vivo results from the literature to construct the aim of comparing the currently known DH treatment approaches with the LOD system, either alone or in different combinations.

Freshly extracted impacted third molars were used here, as in other relevant studies.^5,19,41^ The reason for the selection of impacted teeth was to exclude the possibility of different dentinal tubule diameters and numbers resulting from exposure to the oral cavity, which would have led to external interactions that might have jeopardised the standardisation of tubular integrity in the experimental specimens. The mean values and SD of diameters and numbers of dentinal tubules in citric acid-treated specimens were found to be 2.40 ± 0.06 µm and 154.8 ± 9.9 (control subgroup A); 2.41 ± 0.07 µm and 153.8 ± 7.6 (control subgroup B), respectively, representing the pre-treatment baseline values. It has been reported that immersing in 1% citric acid is the most suitable method for removing smear layer residues without affecting the surface structure.^26^ It was reported that when 1% citric acid is topically applied on a dentin surface only for 1 min, the removal of the smear layer and the exposition of the dentinal tubules occur in the range of 75% to 85%.^26^ In this study, the cementum layer was removed from the underlying dentin surface of the sample teeth 3 mm below the cementoenamel junction, since DH is expected to occur in the cervical area, where dentin may be exposed to the oral environment.^28^

SEM images of the microstructure of experimental specimen surfaces showed that all treatment approaches tested in this study reduced open dentinal tubule diameters and numbers (LOD+Er:YAG > NaF+Er:YAG > LOD > Er:YAG > NaF). SEM images also showed that all treated dentin surfaces were prone to dissolution, allowing the dentinal tubules to re-open (LOD+Er:YAG<NaF+Er:YAG < LOD < Er:YAG < NaF) following acid immersion. However, the LOD+Er:YAG treatment created no open tubules after treatment. and revealed a mean tubule diameter of just 0.09 ± 0.16 µm and a mean tubule number of 0.3 ± 0.48 when immersed in citric acid. This study showed that the LOD+Er:YAG treatment is the most effective procedure of all those examined here for occluding dentinal tubules as well as resisting the 3-h acid attack followed by the applications of NaF+Er:YAG, LOD, Er:YAG laser and NaF gel. Dentin surfaces with the largest number of occluded tubules and greatest diameter reduction revealed a higher resistance to acid attacks. However, the reduction in tubule numbers was not found to be statistically significant in the pairwise comparison of LOD+Er:YAG and NaF+Er:YAG treatments. The fact that these 2 groups – LOD+Er:YAG and NaF+Er:YAG – were equally effective indicates that combined approaches produce better tubule sealing than single methods alone (Tables 1a and 2a). The LOD system applied alone was found to be as effective as when combined with NaF+Er:YAG, and as Er:YAG laser application alone. The DH therapy with Er:YAG laser has been well documented in recent decades. The literature reports that the Er:YAG laser mechanism of action in treating DH is through closure of the dentinal tubules as a result of the evaporation of the dentinal liquid and the precipitation of organic elements and insoluble salts onto the tubular orifices.^46^

The LOD system, on the other hand, generates ultrasonic vibrations at a frequency of 25 kHz, and moves vertically with a horizontal oscillation. Its tip without a true cutting edge sweeps parallel to the root surface in conjunction with an HA-containing polishing fluid. Studies showed that no increase in root-substance removal resulted during root surface debridement when compared to conventional root instrumentation.^8,9^ Patients perceived less pain than in conventional treatments using other hand instruments and ultrasonic devices.^10^ Braun et al^10^ stated that since the LOD system avoids vibrations applied vertically on the root surface and uses HA-containing polishing fluid, the formation of a smear layer on the root surface might cover the dentinal tubules and dull any painful sensations. The authors concluded that LOD instrumentation resulted in a granular layer covering the dentinal tubules. However, they also discovered morphological changes on the root surfaces when the LOD polishing fluid was used with a toothbrush. It was stated that even with manual brushing only, the fluid left a similar granular layer on the root surface covering the dentinal tubules. Therefore, since the polishing fluid is the key factor for sealing the dentinal tubules throughout gentle instrumentation, the LOD system creates an advantage either when treating individual DH symptoms or relieving the pain and discomfort occuring during nonsurgical mechanical debridement.

## Conclusion

The hypothesis of this study was confirmed. LOD alone produced better treatment results than did other singly-applied modalities. The LOD+Er:YAG combination showed the highest treatment effectiveness and acid resistance when compared with the other approaches tested here. Furthermore, LOD alone was also found to be as effective as when combined with NaF+Er:YAG. Further clinical studies are warranted to confirm these in-vitro results suggesting the high potential of LOD for use in DH treatment.

## References

[ref1] Absi E, Addy M, Adams D (1992). Dentine hypersensitivity–the effect of toothbrushing and dietary compounds on dentine in vitro: an SEM study. J Oral Rehabil.

[ref2] Absi E, Addy M, Adams D (1987). Dentine hypersensitivity: a study of the patency of dentinal tubules in sensitive and non-sensitive cervical dentine. J Clin Periodontol.

[ref3] Addy M (1990). Etiology and clinical implications of dentine hypersensitivity. Dent Clin North Am.

[ref4] Addy M, Pearce N (1994). Aetiological, predisposing and environmental factors in dentine hypersensitivity. Arch Oral Biol.

[ref5] Birang R, Yaghini J, Shirani AM (2008). Comparative study of dentin surface changes following Nd:YAG and Er:YAG lasers irradiation and implication for hypersensitivity. J Oral Laser Appl.

[ref6] Brannström M, Linden L, Åstrom A (1967). The hydrodynamics of the dental tubule and of pulp fluid. Caries Res.

[ref7] Braun A, Cichocka A, Semaan E, Krause F, Jepsen S, Frentzen M (2007). Root surfaces after ultrasonic instrumentation with a polishing fluid. Quintessence Int.

[ref8] Braun A, Krause F, Frentzen M, Jepsen S (2005). Removal of root substance with the Vector™-system compared with conventional debridement in vitro. J Clin Periodontol.

[ref9] Braun A, Krause F, Hartschen V, Falk W, Jepsen S (2006). Efficiency of the VectorTM-system compared with conventional subgingival debridement in vitro and in vivo. J Clin Periodontol.

[ref10] Braun A, Krause F, Nolden R, Frentzen M (2003). Subjective intensity of pain during the treatment of periodontal lesions with the Vector™-system. J Periodont Res.

[ref11] Cakar G, Kuru B, Ipci SD, Aksoy ZM, Okar I, Yilmaz S (2008). Effect of Er:YAG and CO_2_ lasers with and without sodium fluoride gel on dentinal tubules: a scanning electron microscope examination. Photomed Laser Surg.

[ref12] Canadian Advisory Board on Dentin Hypersensitivity (2003). Consensus-based recommendations for the diagnosis and management of dentin hypersensitivity. J Can Dent Assoc.

[ref13] Chabanski M, Gillam D, Bulman J, Newman H (1996). Prevalence of cervical dentine sensitivity in a population of patients referred to a specialist periodontology department. J Clin Periodontol.

[ref14] Christgau M, Männer T, Beuer S, Hiller KA, Schmalz G (2007). Periodontal healing after non-surgical therapy with a new ultrasonic device: a randomized controlled clinical trial. J Clin Periodontol.

[ref15] Curtis DA, Lin GH, Rajendran Y, Gessese T, Suryadevara J, Kapila YL (2021). Treatment planning considerations in the older adult with periodontal disease. Periodontology 2000.

[ref16] Demi M, Delmé K, Moor R (2009). Hypersensitive teeth: conventional vs laser treatment part I: Conventional treatment of dentin hypersensitivity. J Oral Laser Appl.

[ref17] Dowell P, Addy M, Dummer P (1985). Dentine hypersensitivity: aetiology, differential diagnosis and management. Br Dent J.

[ref18] Gandara BK, Truelove EL (1999). Diagnosis and management of dental erosion. J Contemp Dent Pract.

[ref19] Gholami GA, Fekrazad R, Esmaiel-Nejad A, Kalhori KA (2011). An evaluation of the occluding effects of Er;Cr:YSGG, Nd:YAG, CO_2_ and diode lasers on dentinal tubules: a scanning electron microscope in vitro study. Photomed Laser Surg.

[ref20] Hoffman A, Marshall R, Bartold P (2005). Use of the Vector™ scaling unit in supportive periodontal therapy: a subjective patient evaluation. J Clin Periodontol.

[ref21] Ipci SD, Cakar G, Kuru B, Yilmaz S (2009). Clinical evaluation of lasers and sodium fluoride gel in the treatment of dentine hypersensitivity. Photomed Laser Surg.

[ref22] Jakubovics NS, Goodman SD, Mashburn-Warren L, Stafford GP, Cieplik F (2021). The dental plaque biofilm matrix. Periodontology 2000.

[ref23] Kumar NG, Mehta D (2005). Short-term assessment of the Nd:YAG laser with and without sodium fluoride varnish in the treatment of dentin hypersensitivity–a clinical and scanning electron microscopy study. J Periodontol.

[ref24] Lan W-H, Liu H-C (1996). Treatment of dentin hypersensitivity by Nd:YAG laser. J Lasers Med Sci.

[ref25] Lopes AO, Aranha ACC (2013). Comparative evaluation of the effects of Nd:YAG laser and a desensitizer agent on the treatment of dentin hypersensitivity: a clinical study. Photomed Laser Surg.

[ref26] McAndrew R, Kourkouta S (1995). Effects of toothbrushing prior and/or subsequent to dietary acid application on smear layer formation and the patency of dentinal tubules: an SEM study. J Periodontol.

[ref27] Naylor F, Corrêaaranha AC, Eduardo CDP, Arana-Chavez VE, Sobral MAP (2006). Micromorphological analysis of dentinal structure after irradiation with Nd:YAG laser and immersion in acidic beverages. Photomed Laser Surg.

[ref28] Orchardson R (1987). Clinical features of hypersensitive teeth. Br Dent J.

[ref29] Orchardson R, Gillam DG (2006). Managing dentin hypersensitivity. J Am Dent Assoc.

[ref30] Powell LV, Gordon GE, Johnson GH (1990). Sensitivity restored of Class V abrasion/erosion lesions. J Am Dent Assoc.

[ref31] Qin C, Xu J, Zhang Y (2006). Spectroscopic investigation of the function of aqueous 2-hydroxyethylmethacrylate/glutaraldehyde solution as a dentin desensitizer. Eur J Oral Sci.

[ref32] Ritter AV, de Dias WL, Miguez P, Caplan DJ, Swift Jr EJ (2006). Treating cervical dentin hypersensitivity with fluoride varnish. J Am Dent Assoc.

[ref33] Schwarz F, Aoki A, Sculean A, Georg T, Scherbaum W, Becker J (2003). In vivo effects of an Er: YAG laser, an ultrasonic system and scaling and root planing on the biocompatibility of periodontally diseased root surfaces in cultures of human PDL fibroblasts. Lasers Surg Med.

[ref34] Schwarz F, Bieling K, Venghaus S, Sculean A, Jepsen S, Becker J (2006). Influence of fluorescence-controlled Er:YAG laser radiation, the Vector™ system and hand instruments on periodontally diseased root surfaces in vivo. J Clin Periodontol.

[ref35] Sculean A, Schwarz F, Berakdar M, Romanos GE, Brecx M, Willershausen B (2004). Non-surgical periodontal treatment with a new ultrasonic device (Vector™-ultrasonic system) or hand instruments: A prospective, controlled clinical study. J Clin Periodontol.

[ref36] Taani SQ, Awartani F (2002). Clinical evaluation of cervical dentin sensitivity (CDS) in patients attending general dental clinics (GDC) and periodontal specialty clinics (PSC). J Clin Periodontol.

[ref37] Tal M, Oron M, Gedalia I, Ehrlich J (1976). X-ray diffraction and scanning electron microscope investigations of fluoride-treated dentine in man. Arch Oral Biol.

[ref38] Ten Cate JM (1999). Current concepts on the theories of the mechanism of action of fluoride. Acta Odontol Scand.

[ref39] Ten Cate JM (1997). Review on fluoride, with special emphasis on calcium fluoride mechanisms in caries prevention. Eur J Oral Sci.

[ref40] Tunar OL, Gursoy H, Ozkan Karaca E, Kuru BE (2021). A comparative evaluation of root surface biomodification with erbium-doped yttrium aluminum garnet laser, ethylenediaminetetraacetic acid gel, and titanium nitride curette: in vitro scanning electron microscope and profilometry analyses. Photomed Laser Surg.

[ref41] Tunar OL, Gürsoy H, Çakar G, Kuru B, Ipci SD, Yılmaz S (2014). Evaluation of the effects of Er: YAG laser and desensitizing paste containing 8% arginine and calcium carbonate, and their combinations on human dentine tubules: a scanning electron microscopic analysis. Photomed Laser Surg.

[ref42] Vieira AHM, Passos VF, de Assis JS, Mendonça JS, Santiago SL (2009). Clinical evaluation of a 3% potassium oxalate gel and a GaAlAs laser for the treatment of dentinal hypersensitivity. Photomed Laser Surg.

[ref43] Visuri SR, Walsh Jr JT, Wigdor HA (1996). Erbium laser ablation of dental hard tissue: effect of water cooling. Lasers Surg Med.

[ref44] Von Troil B, Needleman I, Sanz M (2002). A systematic review of the prevalence of root sensitivity following periodontal therapy. J Clin Periodontol.

[ref45] Wade WG (2021). Resilience of the oral microbiome. Periodontology 2000.

[ref46] Watanabe H, Kataoka K, Iwami H, Shinoki T, Okagami Y, Ishikawa I (2003). In vitro and in vivo studies on application of erbium:YAG laser for dentine hypersensitivity. International Congress Series Elsevier.

[ref47] West NX (2008). Dentine hypersensitivity: preventive and therapeutic approaches to treatment. Periodontol 2000.

[ref48] White DJ, Lawless MA, Fatade A, Baig A, von Koppenfels R, Duschner H (2007). Stannous fluoride/sodium hexametaphosphate dentifrice increases dentin resistance to tubule exposure in vitro. J Clin Dent.

[ref49] Yoshiyama M, Masada J, Uchida A, Ishida H (1989). Scanning electron microscopic characterization of sensitive vs. insensitive human radicular dentin. J Dent Res.

[ref50] Zero DT, Lussi A (2005). Erosion – chemical and biological factors of importance to the dental practitioner. Int Dent J.

